# Who withdraws from caregiver research and why: The Tele‐STELLA experience

**DOI:** 10.1002/alz70858_107227

**Published:** 2025-12-26

**Authors:** Allison Lindauer, Aimee R Mooney, Christina S Zonker, Emily Tupper, Heather Franklin

**Affiliations:** ^1^ Oregon Health & Science University, Portland, OR, USA; ^2^ Oregon Alzheimer's Disease Research Center, Portland, OR, USA; ^3^ OHSU School of Nursing, Portland, OR, USA

## Abstract

**Background:**

The extant literature on who withdraws from caregiver behavioral interventions and why is limited. Here we describe the sample of caregivers who withdrew from Tele‐STELLA (Support via Technology: Living and Learning with Advancing dementia). This telehealth‐based intervention guided family caregivers on strategies to reduce distressing behavioral symptoms of dementia (NIA R01AG067546). The caregivers were enrolled to participate in the intervention; the persons with dementia were enrolled for data collection only.

**Method:**

Caregivers who withdrew were compared to those that did not on variables including age, race, rural, computer use, satisfaction with the study, dementia type and stage, and burden. Categorical variables were analyzed using chi‐squared tests. Continuous variables were analyzed using t‐tests or ANOVA. *p*‐values were calculated to assess statistical significance, with values below 0.05 considered significant.

**Result:**

Fifty‐seven (30.3%) caregivers withdrew from Tele‐STELLA. Severe dementia in care recipients was significantly associated with caregiver withdrawal (*p* = 0.028), and the main reason for withdrawal was the death of the care recipient with dementia (*n* = 19, 33.9%). Death was the leading reason for withdrawal after the active intervention (*p* = 0.027). Caregivers who reported overwhelming responsibilities accounted for 21.1% of the withdrawals (*n* = 11), and this was leading reason during the active intervention (*p* = 0.027). Caregivers who withdrew before the active intervention had the lowest number of adverse events (mean = 0.429), while those withdrawing after the active intervention had the highest number of adverse events (mean = 1.57, *p* < 0.001). Age, race, gender, rural status, computer use confidence, burden, and study satisfaction were not associated with withdrawal.

**Conclusion:**

The findings suggest family caregivers need assistance with behavioral symptom management, and sign up for intervention studies, even at the end of life. They withdrew from Tele‐STELLA due to care recipient death or other adverse events. They did withdraw not due to demographic variables, burden, or dissatisfaction with the study. Participants caring for a family member in the later stages of dementia provide valuable data about their experiences and stressors. Our future work will adjust withdrawal expectations by an additional 10% so we can continue to learn about these families and their needs for support.

